# An assessment of the future impact of alternative technologies on antibiotics markets

**DOI:** 10.1186/s40545-016-0085-3

**Published:** 2016-10-26

**Authors:** Ejike Nwokoro, Ross Leach, Christine Årdal, Enrico Baraldi, Kellie Ryan, Jens Plahte

**Affiliations:** 1 Norwegian Institute of Public Health, Oslo, Norway; 2 Infection Control Program and Division of Infectious Diseases, Geneva University Hospitals and Faculty of Medicine, Geneva, Switzerland; 3 Uppsala University, Uppsala, Sweden; 4 AstraZeneca, Gaithersburg, MD USA

## Abstract

**Background:**

The increasing threat of antimicrobial resistance combined with the paucity of new classes of antibiotics represents a serious public health challenge. New treatment technologies could, in theory, have a significant impact on the future use of traditional antibiotics, be it by facilitating rational and responsible use or by product substitution in the existing antibiotics markets, including by reducing the incidence of bacterial infections through preventative approaches. The aim of this paper is to assess the potential of alternative technologies in reducing clinical use of and demand for antibiotics, and to briefly indicate which segments of the antibiotics market that might be impacted by these technologies.

**Methods:**

An initial mapping exercise to identify the alternative technologies was followed by a review of relevant published and grey literature (*n* = 52). We also carried out stakeholder engagement activities by a round-table discussion with infectious disease specialists and a multi-criteria decision analysis exercise with pharmaceutical industry experts.

**Results:**

Ten alternative technologies were identified and analyzed for their potential impact on the antibiotics market. Of these, rapid point-of-care diagnostics, vaccines, fecal microbiota transplantation, and probiotics were considered to have a “high” or “medium” potential impact over a 10-20 year horizon. Therapeutic antibodies, antibiotic biomaterials, bacteriophages, antimicrobial nanoparticles, antimicrobial peptides, and anti-virulence materials were rated as having “low” potential impact.

**Conclusion:**

Despite the apparent potential of the most promising alternative technologies to reduce demand, that reduction will likely only happen in limited segments of the antibiotics market or, in the case of preventing community acquired streptococcal infections by vaccination, in a low-price generics market segment. Thus, alternative technologies are not expected to represent any disincentive to antibiotics developers. Finally, it is unlikely that alternative technologies will displace the need for new classes, and sub-classes, of antibiotics in the short and medium terms.

## Background

Antibiotic resistance is regarded as a major threat to global public health, to the extent that medicine could be on its way “back to the future” of a pre-antibiotic era [1]. The issue is receiving high-level political attention, with resolutions passed at events such as the G7 Health Ministries summit [2] and the European Parliament [3], the endorsement by the WHO of the AMR Global Action Plan [4], and culminating at the United Nations High-Level Meeting on AMR and the adoption of its declaration by the UN General Assembly [5]. Likewise, in academic circles the issue has been given widespread attention, for instance by the 2015 Lancet Series on *Antimicrobials: Access and sustainable effectiveness* [6–11], the Chatham House Report on *A New Global Business Model for Antibiotics - Delinking Revenues from Sales* [12], as well as the work done by both the Eastern European Group (ERG)[13] and IMS Health [14] on assessing and estimating the parameters relevant for incentivizing antibiotics R&D in the face of the increasing rate of bacterial resistance to existing antibiotics. Several initiatives have been set up to address the issue, such as the Transatlantic Task Force on Antimicrobial Resistance (TATFAR) [15], the UK Review on Antimicrobial Resistance (the AMR Review) [16], which has already issued several reports on different aspects of the antimicrobial resistance challenge [17–22], and the *Driving reinvestment in research and development and responsible antibiotic use* (DRIVE-AB) project [23] funded by Europe’s Innovative Medicines Initiative (IMI) [24].

The work presented in this paper is part of DRIVE-AB, a consortium of 16 public sector partners and seven pharmaceutical companies. DRIVE-AB is tasked with defining responsible use of antibiotics, identifying the antibiotic-related public health priorities, calculating the societal value of having new antibiotics available for these priorities, developing and costing new economic models to promote antibiotic innovation and sustainable use of the resulting, novel antibiotics.

The problem of bacterial resistance to existing antibiotics is exacerbated by declining numbers of multinational pharmaceutical companies that are currently engaged in research and development of new antibiotics, and concurrently, the limited number of new classes of antibiotics in the R&D pipeline [25]. Use of any future novel antibiotics is anticipated to be highly limited in the first few years of launch to maintain their effectiveness, leading to a poor commercial environment and low returns on investment relative to other therapy areas. In short, the market for antibiotics is not sufficiently profitable to incentivize companies to maintain an R&D pipeline that could meet the present and future threat of antibiotic resistance.

Despite these challenges, antibacterial innovation continues through new technologies such as bacteriophages (i.e., viruses that attack and kill specific bacteria) or vaccines. One might wonder if these innovations have the potential to replace antibiotic treatment for certain pathogens if they should become included in future treatment protocols. Several papers, among them Allen et al. [26], Fernebro [27] and, recently an AMR Review report [21] and a review paper by Czaplewski et al. [28], have identified and discussed such technologies, but without explicitly assessing their potential impact on the antibiotics markets. Thus, in this paper we assess the effect that different technologies might have on use and demand for antibiotics in different segments of the antibiotics market, taking the perspectives from industry, clinical practice and health policy research. We also ask whether alternative technologies could potentially counter antibiotic resistance to the extent that they would reduce the need for developing new antibiotics in the short and medium terms.

## Methods

Our research aimed at identifying a range of alternative technologies that can either be used as *substitutive* treatments to antibiotics, or that could dramatically impact the size of a particular antibiotic market segment when used together with antibiotics (so-called *complementary* technologies). We consider substitutive technologies to be any substance, product or technology not classified as a traditional antibiotic that would perform the same task as a traditional antibiotic, i.e. kill or inhibit the growth of bacteria. We limited the study to human medicine only. We assessed which segments of the antibiotics market might be impacted by these technologies and to what extent, within the next 10 – 20 years.

As already indicated, substitutive technologies can potentially replace antibiotics in the treatment of infections [26, 27] and thereby reduce market size, while complementary technologies, such as rapid point-of-care diagnostics (RPOCD), have the potential both to reduce clinical trial costs and facilitate responsible use [29] and thereby can either increase or reduce sales of a specific antibiotic.

Our research design comprises three independent assessment procedures (described in detail below), preceded by an initial mapping exercise to identify the technologies that would be included in the assessments. The three procedures were a literature review, a Multi-Criteria Decision Analysis (MCDA) with industry experts, and a roundtable discussion with infectious disease clinicians. Lastly, building on the output from all these activities, we assessed which segments of the antibiotics market that would likely be most affected by each of the alternative technologies. The following paragraphs explain the initial mapping exercise and the assessment processes in more detail.

### Initial mapping exercise

The initial mapping exercise was an exploratory interactive process within the DRIVE-AB research team to define the scope and to get a sense of the breadth of coverage of technologies to different infections, with the intermediate goal of identifying the technologies to be included in the study. The research team is a multidisciplinary group of professionals with expertise in drug manufacturing, healthcare research, infectious diseases, business modelling, health economics and pharmaceutical policy. This exploratory process included consulting known literature on an ad-hoc basis; a total of eleven sources [30–39] were referenced, of which Fernebro’s review paper [27] was used as a point of departure.

The inclusion criteria were that the technologies should a) kill or inhibit the growth of bacteria (i.e. *alternatives*), or b) enhance the efficiency or effectiveness of traditional antibiotics (i.e. *complementary*). It was also decided to only include technologies that have been subject to significant R&D efforts during the last 20 years; thereby excluding obsolete therapies and purely embryonic technologies.

### Literature review

The aim of the literature review was to review industry pipelines to assess the potential of each technology to deliver products meeting future clinical need. Thus, we defined three criteria: 1) whether there are products in the R&D pipelines for the selected technologies, 2) whether they are in current clinical use, and 3) whether they address identified clinical needs. Clinical needs were defined as the 15 priority pathogens identified by the US Center for Disease Control and Prevention as “urgent threats” and “serious threats” (the three pathogens in the “concerning threats“ category were excluded) [40]. If all three criteria were met, the technology would be considered to have a high potential to deliver products meeting future clinical needs, while if less than three criteria were met the technology would be considered to have a low potential.

In July 2015, two authors (RL and EN) searched the PubMed and Google-Scholar databases using the terms in Table 1 (page 15) as the search terms, e.g. “antibiotic biomaterial” and “therapeutic antibodies”. The yields of each search were in the range between the tens and several hundred thousands. Primary research articles and reviews containing information on the description, strengths and/or weaknesses of the identified technologies were selected. The reference lists of the identified papers were scanned to search for more eligible articles.

**Table 1:** Alternative technologies discussed in this paper

**Antibiotic biomaterials** Antibiotic biomaterials are substances that are added to or form part of implants, prostheses, bandages or other medical devices in order to reduce the risk of bacterial infection. The working principle is that the biomaterial delivers an antibiotic drug over a prolonged time directly at the local site that is a risk of bacterial invasion and infection. There is inconclusive evidence on appropriate and optimal clinical use of this technology, and lack of standardized treatment protocols [85].
**Antimicrobial nanoparticles** Nanoparticles are a drug delivery method that could be applicable for antibiotics. There are multiple working principles, including to improve the solubility of poorly water-soluble drugs; to prolong the half-life of drug systemic circulation by reducing immunogenicity; to release drugs at a sustained rate or in an environmentally responsive manner and thus lowers the frequency of administration; to deliver drugs in a target manner to minimize systemic side effects; and to deliver two or more drugs simultaneously for combination therapy to generate a synergistic effect and suppress drug resistance. There seems to be no nanoparticle based antibiotic therapies in clinical use [37, 106].
**Antimicrobial peptides** These molecules are produced as part of the innate (non-specific) immune system which confers defense against infections without prior exposure to foreign pathogens. The working principle is that the peptides would disrupt cell membranes, with a broad spectrum effect on a variety of microbes, including bacteria. The exact mechanism of action is still unclear. Hence, the potential clinical utility of this technology is yet to be determined [72].
**Anti-virulence materials** Therapeutic agents which target the mechanisms and processes through which microbes cause infection or a pathogenic cascade. In contrast to traditional antibiotics, the working principle is not to kill the pathogen, but to inhibit its capacity to cause illness. In other words, anti-virulence materials could prevent specific bacteria from adhering to human tissue, or inhibit bacterial quorum sensing or secretion of toxins, or make specific bacteria more sensitive to traditional antibiotics. Few, if any, antibacterial candidates have moved beyond animal model studies [107].
**Bacteriophages (including lysins)** Bacteriophages are a type of virus that infects bacteria. The working principle of using bacteriophages as a therapeutic agent is that the phages would infect a pathogenic bacterial cell, whereupon they would replicate to synthesize genome and structural proteins into progeny virions inside the host cell. Finally the new phages would escape by rupturing the bacterial cell wall which results in the death of the cell. The escaping phages would in turn be capable of infecting other bacterial cells. Phages are highly bacteria-specific. This technology is in clinical use in some Eastern European countries, including Georgia, Poland, and Russia [70, 71].
**Fecal microbiota transplantation (FMT)** FMT involves transplantation of feces from healthy donors into the gut of individuals with a gastrointestinal infection or condition. The working principle is that the bacteria from the donor would restore the microbiological environment in the patient’s intestines, thus eliminating the pathogenic bacteria. Clinical trials have demonstrated effect on *Clostridium difficile* infection, but standardized clinical protocols have yet to be developed [65].
**Probiotics** Probiotics are live microorganisms which, when administered in adequate amounts, confer a health benefit on the host. The working principle is similar to that of FMT in that the ingested microorganisms improve the function of the intestinal flora of the patient. Clinical trials studying the effect of probiotics against a variety of conditions have been carried out, including bacterial infections, with diverging results. As with FMT, standardized clinical protocols are not yet in widespread use [108]
**Rapid point-of-care diagnostics** RPOCD are analytical testing performed outside the central laboratory, and can be based on a range of technologies, including antigen based tests, whole genome sequencing, real-time polymerase chain reaction, probe-based assays, bioluminescence real-time amplification, and microarray or micropump technologies. In a clinical perspective, the working principle is to use a device or devices that can be easily transported to the vicinity of the patient, with the benefit of rapid diagnosis and concurrent onset of appropriate treatment. In outpatient settings, RPOCD could provide access to diagnostics in resource constrained settings, or instant diagnosis could save the patient the delay caused by having to pay the clinic an additional visit to receive test results and the appropriate treatment [52].
**Vaccines** A vaccine is a biological preparation that improves immunity to a particular microorganism. A vaccine typically contains an agent that resembles a disease-causing microorganism, and is often made from weakened or killed forms of the microbe, its toxins or one of its surface proteins. The working principle is to stimulate the body's immune system to recognize the agent as foreign, destroy it, and "remember" it, so that the immune system can more easily recognize and destroy any of these microorganisms that it later encounters. Vaccines against a range of different viral and bacterial diseases are in widespread use [30].
**Therapeutic antibodies** Antibodies are synthesized in the human body by the plasma cells as a response to an invading foreign agent. Monoclonal antibodies can be produced in cell culture, but antibodies can also be produced *in vivo* by extraction from blood material, for instance. The working principle of therapeutic antibodies is that when injected into the human body, the antibodies will bind to specific locations on specific microbial cells or proteins, thus facilitating the natural immune system in eliminating that cell or protein. Anti-microbial antibodies fall in two broad categories; those that bind directly to the pathogen, and those that aim to neutralize toxins or other virulence factors. There seems to be no antibodies based antibiotic therapies in clinical use [109].

Given that the aim was to *confirm* R&D activities, clinical practice and priority pathogen targeting, it was decided to limit the reading to the point at which such confirmation had been established, so that additional reading would only add redundancy. For vaccines, this was a particular case in point. A basic search for “vaccines AND antibiotics” in Google Scholar generates around 200,000 results. Furthermore, as a well-developed R&D space with many bacterial vaccines being used in routine healthcare, information on the bacterial vaccine pipeline was identified through a 2013 report issued by the Pharmaceutical Research and Manufacturers of America [41]. Additional literature, i.e. three papers specifically on pneumococal vaccines [42–44] and a public report from the Product Development Partnership PATH [45] were reviewed. This was supplemented by a review of the clinical trial database ClinicalTrials.gov at the point in time of the research (mid-2015) to get an up-to-date picture on the status of vaccines under development, as well as by cross-checking against relevant company websites. However, there was a lack of peer-reviewed publications available on the specific products in the pipeline; perhaps a consequence of the average phase of R&D (typically Phase II) of the bacterial vaccines in the pipeline. For probiotics, eight papers were needed to reach the information saturation point. In total, 52 papers were reviewed.

The review identified 15 companies that had any of the ten alternative technologies on the market, and annual reports (*n* = 13) and press releases (*n* = 2) were obtained and added to the review procedure. We also searched ClinicalTrials.gov, for each alternative technology, which yielded references to 148 relevant trials.

### Multi-criteria decision analysis by industry experts

Following identification of the technologies, we undertook a Multi-Criteria Decision Analysis (MCDA) involving a panel of industry experts to assess whether the technologies have the potential to significantly reduce the demand for traditional antibiotics in the next 10 – 20 years. MCDA involves breaking down an assessment or decision problem into smaller and more manageable questions by which it can be evaluated against a set of predefined criteria. This is a particularly robust method when dealing with complex and fragmented information, such as in this context [46].

The entire research team participated in the design of the MCDA process. In practice, this meant that both the academic partners (who reviewed and analyzed the final MCDA data) and industry partners (who either submitted scores individually or as part of a wider group response) discussed and jointly agreed upfront on the most relevant criteria and on the scoring method [46]. This process was re-stated in an email sent to participants, alongside an MS Excel file to collect scores.

In this case, we broke down the assessment problem into three questions for each selected technology:

1. What is the estimated impact on the demand for antibiotics? Score of 1 means the technology will have little impact on antibiotics demand while score of 3 means the technology will greatly reduce demand for antibiotics.
2. What is the development cost? Score of 1 means the technology will be very costly to develop, while a score of 3 means the technology will have a relatively low cost of development, as compared to traditional antibiotics.
3. What is the expected time for the technology to reach the market? Score of 1 means the technology will be very slow to market, i.e., more than 10 years, while a score of 3 means the technology will be on the market soon, i.e., less than five years.

The three criteria were given equal weights, and it is evident that the two latter questions somehow modify the assessment provided in the first question. The experts also used a 1-3 scoring system to indicate how confident they were in their assessment of each of the ten technologies (please see summary of results in Table 2 on page 17).

**Table 2:** Overview of assessments of technologies

Assessment process:	Literature review			Industry expert MCDA		Clinicians roundtable discussion	Summary score
Assessment parameter:	Potential to deliver products			Potential to reduce demand		Potential to impact on clinical use	Overall potential
Assessment criteria, or score:	In clinical use	Products in pipeline	Targets priority pathogen (CDC list)	Score (max 27, min 6)	Assessment*	May reduce antibiotics demand	
Rapid point-of-care diagnostics	Yes	Yes	Yes	20.0	High	Yes	High
Vaccines	Yes	Yes	Yes	14.8	High	Yes	High
Probiotics	No	Yes	Yes	15.4	High	Yes	Medium
Fecal microbiota transplantation	No	Yes	Yes	15.0	High	Yes	Medium
Therapeutic antibodies	Yes	Yes	Yes	15.1	High	No	Low
Antimicrobial peptides	Yes	Yes	Yes	11.4	Low	No	Low
Antibiotic biomaterials	Yes	No	n.a.	12.4	Low	Yes	Low
Antimicrobial nanoparticles	Yes	Yes	No	8.0	Low	No	Low
Anti-virulence materials	No	No	n.a.	12.0	Low	No consensus	Low
Bacteriophages (and lysins)	No	Yes	Yes	8.8	Low	No	Low

Legend: “Yes” means a response in the affirmative for the criterion in any given the column heading, whereas “No” means that the criterion was not fulfilled

*High potential technologies are those with a total score greater than the median of the total scores of the entire data set (13.4), where total score = (Time + Demand + Cost) x Confidence

The experts who contributed scores were recruited from among the DRIVE-AB consortium pharmaceutical industry partners from the European Federation of Pharmaceutical Industries and Associations (EFPIA). Five major pharmaceutical companies were represented (Roche, AstraZeneca, GSK, Pfizer and Astellas).

To avoid potential bias, the three members of the research team who were also industry expert participants in this MCDA assessment process did not have access to the outputs of the literature review and clinician roundtable before they participated in the MCDA assessment. Finally, the academic partner responsible for the analysis summarized the scores and provided a summary analysis to the research team. The research team reviewed the summary analysis and signed off the final result of the MCDA process.

### Clinician roundtable discussion

The third assessment procedure was an expert panel roundtable discussion involving a group of three infectious disease physicians to assess the potential clinical utility of the alternative technologies and the potential of each of the technologies to significantly reduce or otherwise impact on the use of traditional antibiotics in routine clinical practice in priority disease areas. Apart from their infectious diseases specialties, the three panelists together held specific expertise in conducting clinical trials, *inter alia* on both probiotics and FMT, and they are all based at the University Hospitals of Geneva (UHG) in Switzerland. UHG is one of the participating entities in the DRIVE-AB project, but none of the three doctors are a part of the research team for this work stream. The aim of the roundtable discussion was to assess the ten technologies from a clinical perspective in terms of future (potential) clinical utility, thus triangulating the results from the literature review and the MCDA exercise

The process took the form of *focus group discussions* [47] and *expert knowledge elicitation* [48]. This roundtable discussion research design aimed at facilitating *on site* generation and exchange of opinions in a critical discussion.

The panelists were forwarded a list of eleven selected alternative technologies (FMT and microbiome therapeutics were presented as two separate categories on this occasion), and a guide suggesting the questions to be addressed. One member of our research team (RL) provided a detailed briefing on the basic objective, approach, and what input was needed, and subsequently moderated the discussion while other team members listened in remotely. Although there was no request made to reach consensus, the panelists’ opinions tended to converge on most of the subject matter.

The panel carried out an assessment of the future potential clinical utility of the alternative technologies, based on whether the technology is likely to enter routine clinical practice in the next 10 – 20 years to an extent that will significantly reduce or otherwise impact the use of traditional antibiotics. The roundtable was also tasked with determining whether the technology can be a substitute or a complement to antibiotics, whether the technology has a broad or narrow bacterial spectrum potential, and whether the use of the alternative technology depends on the availability of appropriate diagnostics.

### Similar criterion

Our design includes the redundancy of using the criterion of “demand impact” in the MCDA and the criterion “impact on use” by the clinicians roundtable, where the distinction between “demand” and “use” is somewhat subtle. Admittedly, an alternative technology may impact the antibiotics markets without having to target clinical needs; for instance, in theory a probiotic treatment, if effective, could outcompete a perfectly effective antibiotics based on lower price. Nevertheless, we are assuming that for the most part clinical need is a prerequisite for commercial viability, although not all clinical needs are expressed as effective demand.

## Results

### Identified alternative technologies

Table 1 lists the ten alternative technologies that were selected for assessment for this paper by the initial mapping exercise. RPOCD and some anti-virulence materials are *complementary*, while the remaining eight technologies are mainly *substitutive*.

### Synthesis of assessments of alternative technologies

The results of the literature review, MCDA-round and the clinical roundtable are given in Table 2. The summary score indicates the potential of each technology in bringing products to the market that are adopted in clinical use to the degree that it has an impact on antibiotics demand. Vaccines and diagnostics are rated as overall “high potential”, having met all basic criteria, and FMT and probiotics are rated “medium potential”. The remaining six technologies were rated as “low potential”.

First, we would remark that the global USD 40 billion antibiotics market is in reality a patchwork of fragmented and partially overlapping markets, defined and delimited by a multitude of different pathogens and diseases, commonly with different treatment protocols and guidelines in different countries and regions. Resistance patterns, and thus antibiotic use patterns, also vary geographically. In the following, it will be demonstrated that certain markets stand to be affected more than others by the different substitutive and complementary technologies. The impact on the antibiotics markets could also be analyzed in terms of whether the technologies affect the use of first, second or third-line therapies and by extension, their impact on generics and novel antibiotics, respectively.

The following paragraphs present a synthesis of the assessments of how each of the ten technologies could be expected to impact the different market segments for antibiotics. The focus is on the four “high” and “medium” potential technologies (RPOCD, FMT, probiotics and vaccines). We found it to be outside of the scope of this work, and possibly simply overly speculative, to model the future impact of prospective alternative technologies on future antibiotics markets in quantitative terms. Hence, the following assessment is made in terms of qualitative denominators, such as “widespread use” and “limited impact”.

### Rapid point-of-care diagnostics (RPOCD)

Despite being “just” complementary and not substitutive, based on the results of the MCDA and clinician’s roundtable, RPOCD is the technology expected to have the most profound effect on antibiotics demand. Diagnostics can be developed at relatively low cost, and could be available for routine use in the next five years, or less. However, the extent to which RPOCD has a sustained impact on antibiotic demand is unclear, and in practice may ultimately reflect the level of specificity of test available, and its ability to effectively integrate into health systems for routine use.

First, widespread use of rapid diagnostic tests in community settings could reduce inappropriate and unnecessary use of antibiotics against non-bacterial infections. This would reduce the demand for many broad-spectrum antibiotics, but to a varying degree depending on the normal consumption patterns in each market. Second, RPOCD could be an effective tool to support the diagnosis of severe infections. However, despite the utility of diagnostics in improving clinical practice in hospital settings, empiric treatment is expected to remain a widespread approach, and diagnostics are not expected to significantly reduce the demand for antibiotics in any specific disease area. Rather, RPOCD are expected to impact antibiotic markets by allowing more targeted therapy – not by reducing total antibiotics demand but by reducing demand of broader spectrum antibiotics to be replaced by more narrow spectrum antibiotics depending on microbiologic environment [49–53].

The MCDA pointed out low development costs and short time to market as factors that could increase the impact of RPOCD on antibiotic stewardship, whereas the clinicians pointed out that there remain basic implementation challenges with RPOCD; diagnostics can be costly in clinical use, especially in a context where clinicians may not utilize the available diagnostics results in making clinical decisions, and there could be disagreements within hospitals on the deployment of resources; microbiologists may tend to prefer deployment of testing in the laboratory instead of remotely within an Intensive Care Unit, for instance.

It should be added that the wider benefit of diagnostics could potentially reduce the costs of running clinical trials for new antibiotics. Moreover, very accurate diagnostic tests would be needed for some other technologies considered in this paper, such as bacteriophages. Cut short, diagnostics are of great utility in improving community-based treatment, facilitating rational prescribing and reducing R&D costs, but will likely not reduce demand for antibiotics in hospital settings.

### Vaccines

Vaccines*,* rated as “high potential”, are already a well understood and widely used technology, and both large pharmaceutical multinationals and small to medium-sized companies are active in this space. The findings of the MCDA suggest that vaccines could have a medium impact on antibiotic demand, are of medium cost to develop, and would take a medium time overall, of around ten years, to become available in the market.

Priority pathogens currently targeted by vaccine R&D pipelines are *Neisseria gonorrhea, Acinetobacter, Escherichia coli, Staphylococcus aureus, Pseudomonas aeruginosa* and *Clostridium difficile*. Generally, there remain some issues to be resolved in terms of identifying and immunizing the target populations, in particular for S. *aureus*, given the challenge of defining any definite risk groups for this pathogen. A vaccine against C. *difficile* would mainly reduce the demand for vancomycin and metronidazole, while the current toolbox for treating different P. *aeruginosa* infections encompasses several handfuls of different antibiotics.

The MCDA experts provided diverging scores on vaccines, which may partly reflect the diverse potential of vaccines. Some vaccines could be widely adopted to address a narrow segment of the market, leading to a small overall impact on antibiotic demand, for instance by targeting small, high-risk populations such as those entering hospital for elective surgery and those living in long-term residential care. This reflects the expected market of bacterial vaccines in the pipeline. In contrast, pneumococcal vaccine, which is the focus of our literature review of vaccines, has been rolled out globally in childhood immunization programs, including in low-income countries, and has had a significant impact on bacterial pneumonia caused by *Streptococcus pneumonia* [42–44].

Furthermore, major questions remain about how new bacterial vaccines can be deployed for small populations in a cost-effective way. This hints at a need to consider alternative reimbursement mechanisms for vaccines also to create further R&D incentives [22]. Overall, even though vaccines may be widely adopted, they would likely impact on a narrow segment of the antibiotics market, leading to a small overall impact on antibiotic demand. Vaccines will not eliminate the need for new antibiotics, given that it is impossible or unfeasible to successfully cover all risk groups with this preventive measure.

### Probiotics

The overall rating of probiotics was “medium potential”. This rating reflects this technology fulfilling all criteria outline in Table 2, with the exception of existing clinical use. The evidence base from the literature is mixed, with diverging opinions on the efficacy of probiotics to treat bacterial infections [34, 54–60]. The MCDA results and the clinician roundtable generated diverging views, with the clinical view that there is a lot of untapped potential in probiotics, in particular for gastrointestinal infections.

The pathogen target for most probiotics R&D and clinical protocols is *C. difficile*, but probiotics also are used to prevent and treat antibiotic associated diarrhea, ulcerative colitis and Crohn’s disease. As yet, there remains a lack of robust evidence for the deployment of probiotics. This is also in the context of strong clinical evidence for alternative approaches, e.g. FMT, for treating *C. difficile*. While in the MCDA analysis probiotics scored high on time to market and cost, but low on impact on demand, the clinicians had comparably high hopes for future probiotic therapies, leading to a contrasting viewpoint on the potential impact on demand.

The literature review shows that additional R&D targets for probiotics are decolonization or inhibition of colonization of *Klebsiella pneumoniae* and *Streptococcus pneumoniae* [34, 54–60]. Thus, given the low cost of probiotics, widespread application could have an impact on the generic antibiotics used to treat community acquired pneumonia [61]. All in all, the total impact on antibiotics markets is mixed, at least in the short or medium-term, although the longer-term potential of probiotics could be more pronounced for specific priority pathogens.

### Fecal microbiota transplantation

Although FMT scored negatively on the criterion “In clinical use” in the literature review, one should bear in mind that several recent clinical studies show strong efficacy for *C. difficile* infections [62, 63]. Ongoing research targets a number of other bacteria and conditions other than gastrointestinal [62, 64–66]. In the MCDA, this technology scored high on short time to market, average on cost, and low on impact on antibiotics demand. The clinicians pointed out major issues for FMT in terms of designing an operational approach for FMT treatment in a local hospital, e.g., creating donor banks, training staff to manage the banks and to deliver the treatment efficiently and effectively. In many contexts, specifically low income countries and other environments with weak health systems, the likelihood of being able to maintain donor banks is low.

More broadly, microbiome therapeutics were viewed by clinicians as a high potential area, with potential to significantly reduce antibiotic use over time through “secondary prevention“, which could also reduce carriage of resistant pathogens through decolonization of pathogens. Overall however, given the narrow application for this technology, the impact on antibiotics markets will likely be very limited.

### The remaining, “low potential” technologies

The following six technologies were regarded as having a low potential to impact on antibiotics demand, mainly due to their limited clinical utility. *Therapeutic antibodies* have been successfully applied in cancer treatment, and raxibacumab is approved for the treatment of patients with inhalational anthrax in combination with appropriate antibacterial drugs. Historically, serum therapy has been used to treat viral and bacterial infections. The main priority pathogen targets are *C. difficile* and *S. aureus*. The main limitation is that this technology is expensive to develop, and given the high specificity of this technology and the concurrent small patient populations, its impact on antibiotics markets will likely be very limited [67–69].

*Bacteriophages* are not currently in use, except for in the few national territories mentioned in Table 1, and the main obstacles to this technology are a lack of a clear regulatory framework, possible lack of patentability, and that the specificity of bacteriophages may require development of unique cocktails for each patient, which in turn implies a dependency on a good diagnostic test to support the selection of treatment “cocktail” [70, 71].

The MCDA analysis generated a range of views on the likely impact of bacteriophages on antibiotic demand, however even where medium to high impact may be possible, this would be predicated on access to the necessary tools to complement bacteriophages (e.g. RPOCD), and the operational capacity to develop phage cocktails. There was general consensus from the MCDA that bacteriophages appear to be relatively expensive to develop (and to deploy on a case-by-case basis), and basic market barriers surrounding regulation suggest that it will take a number of years before this technology is more viable for wide scale deployment. Existing barriers in clinical trial guidelines, a lack of strong efficacy data, and basic operational concerns led the clinicians to remain sceptical about whether bacteriophages could be offered on an empirical basis to sufficiently impact antibiotic demand.

Use of *Antimicrobial peptides* will likely be limited due to their toxicity profile and cost [72–76], although there are numerous attributes of peptides, including a broad application potential and the potential to deploy peptides as a combination therapy. *Antimicrobial nanoparticles* have led to enhanced antibiotic formulations rather than their replacement [77–83].

*Antibiotic biomaterials* have a very narrow scope for application as they are used mainly as wound dressings and as part of implants [84–88]. The literature review identified no *anti-virulence materials* in the R&D pipeline [89–93]. Additionally, this technology is complimentary to antibiotics, and would have little impact on antibiotics demand.

## Discussion

A recent paper [94] that was published after our research activities were finalized concludes that many alternative technologies are facing challenges related to several factors: small markets due to their narrow (even strain specific) spectrums; the need for simultaneous use of both antibiotics and diagnostics; uncertainty as to future rate of resistance development; and the fact that many of them are still far from entering clinical use.

Similarly, in another recent review paper on alternatives to antibiotics [28], the authors conclude that antibodies, probiotics and vaccines are most advanced, and that such therapies targeting *C. difficile*, *P. aeruginosa*, *S. aureus* will likely enter the market. Yet, traditional antibiotics will still be needed as the major antibacterial defense, not least because many alternative technologies have a much narrower or more pathogen specific bacterial spectrum. The authors of this paper also pointed out that in order to have a significant clinical impact, alternative technologies would need increased funding in the order of US$ 2.1 billion over the next ten years.

Another paper published while writing up our research results is the AMR Review report on “Vaccines and alternative approaches” [21]. As indicated by the title, vaccines were identified as the most promising technology for reducing antibiotics consumption, but new demand-oriented reward mechanisms are recommended to boost the pipelines of bacteriophages (and lysins), antibodies, probiotics, peptides and immune stimulating technologies. The report points at narrow bacterial spectrum and the novelty of the products as seen from regulators’ and clinicians’ point of view as potential obstacles to development and clinical implementation. In our view, although drug regulations were not cited as barriers to development of alternative technologies, ongoing work for developing new regulatory pathways for antibacterial drugs [95] is highly welcome.

Both these contributions, while complementary to our work in many respects, align with some of our main results. Most strikingly, they rank vaccines and probiotics (including FMT) as the most promising of the alternative technologies, while simultaneously pointing out that the narrow bacterial spectrums of many of the alternative technologies will likely limit their impact. Diagnostics were not covered by the Czaplewski et al. paper, but the fact that the AMR Review dedicated a separate report to diagnostics [18] speaks for the importance assigned to this technology by that commission.

### Limitations of this study

The extent to which alternative technologies can impact on the use of and demand for antibiotics depends on a host of factors, including, but not limited to, the availability of knowledge from basic science, the supply of competent labor, investment decisions in private companies, the existence of appropriate regulatory pathways, reimbursement policies and decisions, inclusion in clinical guidelines, and ultimately, the extent to which they are efficiently manufactured and distributed and actually applied in clinical practice [96, 97]. Several barriers to introduction of new technology have been identified in the literature; conservative mentality and professional resistance [98], innovation-unfriendly accounting systems (e.g. DRGs; diagnosis-related group) and complex purchasing procedures [99, 100], and barriers within single hospitals [101], such as lack of motivated champions, power shifts from clinicians to administrators [102–104] or limited educational materials supporting implementation [105]. It was considered beyond the scope of this study to further identify and analyze barriers and bottlenecks to introduce and implement the alternative technologies.

One potential limitation is that we are limited to discussing technologies that have been published or that were familiar to the members of the research team and the participating industry and clinical experts. However, the broad composition of the expert panels and the triangulation of methods served to diminish this potential bias. Throughout the process of developing this paper no technologies other than the ten (Table 1) were identified that met our inclusion criteria.

Another potential limitation was the make-up of the panels. The panels could have represented a broader set of stakeholders, such as health technology assessment agencies (HTAs) or other payers. HTA agencies and payers have an indirect impact on the antibiotic market through reimbursement decisions. However, the purpose of the clinicians’ expert roundtable discussion and the industry representatives’ MCDA was specifically to utilize the specific expertise held by these two stakeholder groups on the demand and the supply side of antibiotic markets, respectively. We believe the triangulation of the results of these two processes with the literature review reduced the risk of stakeholder bias in our final results.

## Conclusion

Despite expectations of widespread use of some the *complementary* and *substitutive* alternative technologies, specifically the “high potential” vaccines and diagnostics and the “medium potential” FMT and probiotics, the impact on the demand for antibiotics in the next 10 to 20 years can be expected to be limited. This is mainly due to the limited range of pathogen targets of the technologies – three of the four “high” or “medium” potential technologies have *C. difficile* as their main target pathogen – and the relatively small patient populations associated with these pathogens. In the one case of a large patient population – community acquired streptococcal infections – the antibiotic market segment likely to be impacted is that for low-priced generics.

None of the technologies should be expected to make any antibiotics redundant, as growing resistance will likely continue to reduce the effectiveness of our current drugs. Alternative technologies, despite all their potential to improve therapies and treatment protocols, will not displace the need for new classes, and sub-classes, of antibiotics. Even though alternative technologies do not contribute to undermining the commercial viability of such novel antibiotics in the near future, new economic models to incentivize increased antibiotics innovation are needed, as mandated to the DRIVE-AB project to propose. Additionally, alternative technologies are needed and their R&D should be encouraged and supported, as recommended by both the AMR Review [21], Czaplewski et al. [28], and Hauser et al. [94], since they help combat antibiotic resistance and facilitate sustainable use of existing and new antibiotics.

## Abbreviations

AMR: antimicrobial resistance
CDC: US Center for Disease Control and Prevention
DRG: diagnosis-related group
DRIVE-AB: Driving reinvestment in research and development and responsible antibiotic use
EFPIA: European Federation of Pharmaceutical Industries and Associations
ERG: Eastern Research Group
FMT: fecal microbiota transplantation
IMI: Innovative Medicines Initiative
MCDA: Multi-Criteria Decision Analysis
MS: Microsoft
R&D: research and development
RPOCD: rapid point-of-care diagnostics
TATFAR: Transatlantic Task Force on Antimicrobial Resistance
UHG: University Hospitals of Geneva
WHO: World Health Organization
